# Death of Sir James Y. Simpson, M. D., of Edinburgh

**Published:** 1870-05

**Authors:** 


### Death of Sir James Y. Simpson, M. D., of Edinburgh.

The sad intelligence of the death of Prof. James Y. Simpson, M. D., was received at Washington, shortly after the sessions of the American Medical Association had closed, from a cable dispatch to Dr. H. R. Storer. A full account of a meeting of the physicians of the United States, at which eulogistic addresses were made, and by which appropriate resolutions were adopted, is now in waiting for the press, and will be presented to our readers in the June number of the *Journal.*

